# Challenging Diagnosis of Addison's Disease Presenting with Adrenal Crisis

**DOI:** 10.1155/2021/7137950

**Published:** 2021-10-11

**Authors:** Ni Wayan Wina Dharmesti, Made Ratna Saraswati, Ketut Suastika, Wira Gotera, I Made Pande Dwipayana

**Affiliations:** ^1^Resident: Internal Medicine Department, Faculty of Medicine Udayana University, Sanglah Hospital, Denpasar, Bali, Indonesia; ^2^Endocrine and Metabolism Division, Internal Medicine Department, Faculty of Medicine Udayana University, Sanglah Hospital, Denpasar, Bali, Indonesia

## Abstract

Primary adrenal insufficiency, also known as Addison's disease, is a rare but potentially fatal condition resulting from the failure of the adrenal cortex to produce glucocorticoid and/or mineralocorticoid hormones. Unfortunately, the clinical manifestation of primary adrenal insufficiency is not specific and often progresses insidiously, resulting in late diagnosis, or in severe cases, life-threatening circulatory collapse. Adrenal insufficiency should be considered in patients with unexplained vascular collapse. We report the case of a woman who presented to the emergency ward with unexplainable shock that was later diagnosed as adrenal crisis due to Addison's disease. The presence of hyperpigmentation in patients with rapid progression of adrenal insufficiency suggests the diagnosis of Addison's disease presenting with adrenal crisis.

## 1. Introduction

Addison's disease is characterized by the failure of the adrenal cortex to produce sufficient glucocorticoids and/or mineralocorticoids (primary adrenocortical insufficiency) to meet metabolic needs because of the destruction or primary dysfunction of the adrenal glands. Decreased secretion of glucocorticoid and/or mineralocorticoid hormones in primary adrenal insufficiency leads to decreased negative feedback to the pituitary gland, which in turn results in increased secretion of adrenocorticotropin hormone (ACTH), which differentiates it from secondary adrenocortical insufficiency (deficiency of ACTH secretion) [1–3]. Addison's disease is a rare disorder with a prevalence of 35–140 cases per million people and an incidence of 4 per million people per year in Caucasian populations [4, 5]. Autoimmunity (autoimmune adrenalitis) accounts for 90% of primary adrenal insufficiency cases reported in western countries [1, 2, 6]. Autoimmune adrenalitis may present as a part of the autoimmune polyglandular syndrome (APS) or as an isolated illness [4, 7]. There is a slight female predominance in the case of autoimmune adrenalitis, with a 2 : 1 female to male ratio [1]. Other conditions that may cause primary adrenocortical insufficiency in addition to autoimmunity include infections, genetic disorders, malignancy, medication, and critical illness.

Primary adrenal insufficiency is a potentially life-threatening condition that demonstrates the vital role of glucocorticoid and mineralocorticoid hormones in maintaining the homeostasis of energy, electrolytes, and body fluids [2]. However, clinical symptoms and signs are usually nonspecific, thereby posing a challenge to diagnosis and, consequently, to providing appropriate treatment. Acute adrenal crisis represents acute adrenocortical insufficiency, with prominent features of hypotension and shock that may rapidly lead to death if patients are left untreated [2]. With the aim of increasing awareness and knowledge of Addison's disease, we report the case of a 27-year-old woman who presented to the emergency ward with shock, which was later found to be caused by primary adrenal insufficiency.

## 2. Case Presentation

A 27-year-old woman was referred to the emergency ward of Sanglah Hospital with a diagnosis of shock caused by suspected hypovolemia or septic shock, acute gastroenteritis with dehydration, suspected autoimmune disease, and suspected pneumonia. The patient had fever for 7 days before admission, accompanied by a productive cough with yellowish phlegm, but no shortness of breath. She had nausea, vomiting, and diarrhea before the onset of fever. Diarrhea was defined as watery stool (without blood) at least three times per day. The patient also reported loss of appetite and loss of body weight (approximately 10 kg within 2 months). The patient had an irregular menstrual cycle since the age of 17 years, and the last menstruation was 18 months ago. She reached menarche at the age of 13 years with a normal growth of breasts and pubic hair. A history of chronic cough, diabetes, or cancer was ruled out. A family history of the same conditions was also excluded. The patient was not taking any regular medication, except for menstruation pills prescribed by her obstetrician several times. The patient was hospitalized for 3 days in another private hospital before admission to Sanglah Hospital and was treated with fluid resuscitation with 1000 ml of crystalloid, intravenous cefoperazone, intravenous paracetamol, intravenous ondansetron, and norepinephrine drip 0.1 mcg/kg BW/h. The patient was unmarried and worked as an employee in a private company.

**Figure 1:**
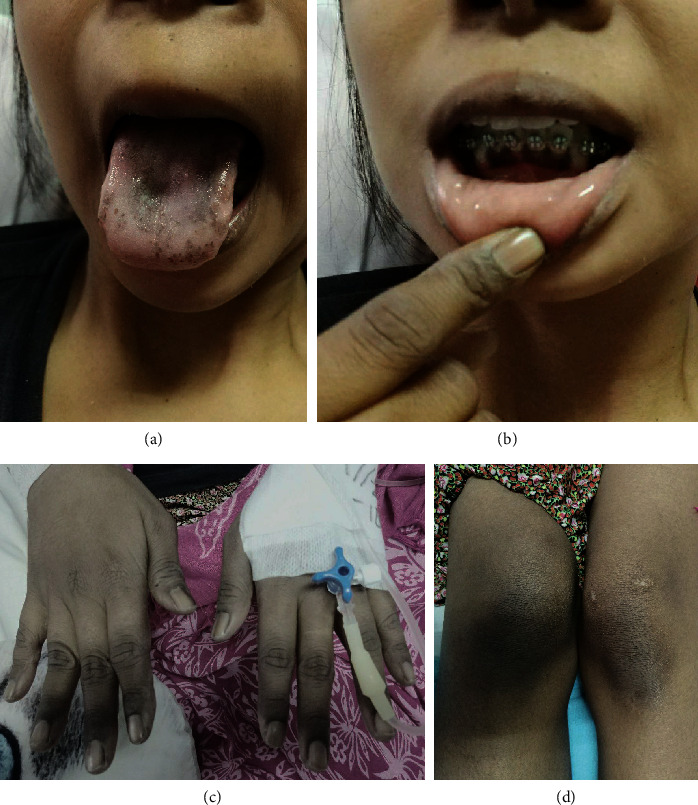
Hyperpigmentation on skin and mucosa: (a) tongue, (b) gum, (c) hands, and (d) knee.

The patient appeared very ill and somnolent upon her first presentation to the emergency ward. Her blood pressure was 90/70 mmHg (on continuous infusion of norepinephrine), heart rate was 108 beats per minute, respiration rate was 22 breaths per minute, and axillary temperature was 37°C. Physical examination revealed a whitish plaque on the tongue, hyperpigmentation of the gingival mucosa, and bilateral skin hyperpigmentation of the interphalangeal joints of fingers and toes as well as knees; her extremities felt cold and moist with a capillary refill time of 2–3 s. No abnormal breath sounds were detected on lung auscultation, and abdominal examination findings were normal (Figure 1).

Laboratory results from a previous hospital showed a high leukocyte count (20.4 × 10^3^ *μ*L) with lymphocytosis, normal hemoglobin and platelet levels, increased serum creatinine level (1.2 mg/dL), and slightly decreased sodium (134 mmol/L), potassium, and blood glucose level both within normal limit. Serial complete blood count examination showed a decrease in leukocyte count (10.6 × 10^3^ *μ*L) before the patient was referred to our hospital. We repeated the examination in the emergency ward and found no abnormalities in complete blood count and levels of serum creatinine, random blood glucose, or liver enzymes. Blood gas analysis showed respiratory alkalosis with a pH of 7.47, pCO₂ of 22.2 mmHg, slightly decreased sodium level (132 mmol/L), and a normal potassium level (4.87 mmol/L). Urine examination and chest radiography were performed to determine possible sources of infection, and both showed normal results. Feces examination could not be performed because the patient did not pass stool while at our hospital. A pregnancy test was performed using a urine sample, and the result was negative.

Our initial assessment was acute diarrhea with possible bacterial or viral causes, severe community-acquired pneumonia with sepsis and septic shock, and secondary amenorrhea. An immunocompromised state was also suspected in our case with a differential diagnosis of systemic lupus erythematosus and human immunodeficiency virus (HIV) infection. Fluid resuscitation was continued in the emergency ward by administering 1000 ml of crystalloid and 0.9% NaCl at 30 drops/min. Antibiotic treatment was also continued with intravenous cefoperazone (1 g every 12 h), intravenous levofloxacin (750 mg every 24 h), oral fluconazole (150 mg every 24 h) to treat oral candidiasis, and continuous infusion of norepinephrine (0.1 mcg/kg BW/h). To determine the cause of amenorrhea, the Obstetric-Gynecologic Department was consulted. However, no abnormality or signs of pregnancy were noted on gynecologic examination. The test results for hepatitis B virus and HIV infection were nonreactive.

The patient's symptoms of nausea, vomiting, weakness, and fever did not improve with the initial treatment. Moreover, despite fluid resuscitation, blood pressure needed to be maintained by administering a continuous infusion of norepinephrine. There was an improvement in the appearance of the whitish plaque on the tongue after 4 days of fluconazole and a hyperpigmentation plaque appeared on the tongue, as shown in Figure 1(a). Following the observation of generalized hyperpigmentation on the skin and mucosa, Addison's disease was suspected, which warranted the evaluation of morning cortisol level. The ACTH level could not be examined because this test is not available in Indonesia. On the following day, morning cortisol hormone level test revealed a very low level (0.84 *μ*g/dL; normal range: 4.3–22.4 *μ*g/dL). The patient was diagnosed as having Addison's disease on day 6 of hospitalization in our hospital, followed immediately by administering intravenous infusion of hydrocortisone (100 mg over 30 min, every 24 h). Abdominal ultrasound was performed to further evaluate the possible underlying conditions, and we found no enlarged lymph nodes, no intra-abdominal masses, and no adrenal gland calcifications. Antibiotic and norepinephrine drip was discontinued 2 days after starting intravenous hydrocortisone, as the patient showed marked clinical improvement, and we found no pathogenic microorganisms in blood and sputum cultures. Repeated examination of sodium and potassium levels, while administering intravenous hydrocortisone, showed that the values were within normal limits. Therefore, we switched to oral hydrocortisone after 7 days of intravenous preparation. The oral dose was started with 20 mg of hydrocortisone in the morning and 10 mg in the afternoon, after which the patient was discharged from hospital. The oral dose was maintained for one month, during which the patient reported disappearance of weakness and return of appetite. The patient also gained her weight and evaluation of electrolytes level was normal. Therefore, we slowly decreased the dose in the second month to 20 mg hydrocortisone taken once daily in the morning.

## 3. Discussion

Primary adrenal insufficiency poses a diagnostic challenge due to its rare incidence and its nonspecific clinical manifestations, which often develop slowly over a long period of time that may cause delay in diagnosis or patients presenting with acute life-threatening condition [3, 8]. Clinical pictures of primary adrenal insufficiency correlate closely with the degree of adrenal gland destruction and is classified as chronic primary adrenal insufficiency and acute adrenal crisis [1]. Patients with chronic primary adrenal insufficiency may show signs and symptoms of hyperpigmentation, weakness and fatigue, loss of body weight, loss of appetite, gastrointestinal disturbances, hypotension, salt craving, and postural symptoms. Hyperpigmentation of the skin and mucosal membrane is an early and classical manifestation of Addison's disease that results from increased ACTH levels; consequently, it does not appear in secondary adrenal insufficiency. Hypotension is present in 90% of the cases and is accompanied by orthostatic symptoms, which in severe chronic cases or acute illness can develop into shock. Amenorrhea is common in Addison's disease due to weight loss, chronic illness, or primary ovarian failure due to polyglandular autoimmune syndrome [1]. Our patient was admitted with nonspecific symptoms of fever, weakness, lethargy, nausea and vomiting, and refractory shock despite the continuous infusion of norepinephrine. Before admitted to the hospital, the patient was complaining of nonspecific symptoms such as loss of appetite, weight loss, and irregular menstruation. The presence of skin and mucosal hyperpigmentation was noticed while the patient was admitted in our hospital and this was the suggestive sign of primary adrenal insufficiency in our patient. However, the diagnosis of primary adrenal insufficiency in our case was suspected after six days of hospitalization after the hyperpigmentation on oral mucosa was clearly seen as the oral thrush was improved.

Acute adrenal crisis, or simply adrenal crisis, is marked by life-threatening, rapid clinical deterioration in patients with adrenal insufficiency and hypotension (systolic blood pressure < 100 mmHg) [9–13]. Adrenal crisis is associated with several risk factors such as chronic adrenal insufficiency, infection, trauma, or surgery [10, 14, 15]. It might appear as the first manifestation of adrenal insufficiency in around 8 per 100 patients with adrenal insufficiency per year, but it is often diagnosed late due to its nonspecific symptoms [16]. Other clinical symptoms that may appear are fever, dehydration, nausea and vomiting, anorexia, weakness, hypoglycemia, and loss of consciousness [1]. Since hypovolemic shock is a common presentation, adrenal insufficiency should be considered in patients with unexplained vascular collapse. The presence of hyperpigmentation of the skin and mucosa in patients with unexplained vascular collapse suggests the diagnosis of primary adrenal insufficiency [1]. Rapid clinical deterioration and refractory shock that did not respond to norepinephrine infusion likely signaled an adrenal crisis in our patient. The adrenal crisis in our case might have been precipitated by lung infection, although no pathogen was found in sputum or blood cultures probably because the culture specimen was taken after several days of antibiotic administration.

The diagnosis of primary adrenal insufficiency is based on a low level of morning (basal) cortisol hormone (either serum or plasma), which is confirmed by low levels of stimulated cortisol by performing corticotropin stimulation test. Primary adrenal insufficiency is also suspected when the basal cortisol level is less than 140 nmol/L (5 *μ*g/dL) and the concentration of plasma ACTH is increased by 2-fold from the upper limit of normal [2]. Corticotropin stimulation test, also known as cosyntropin test or ACTH test, is considered the gold standard for the diagnosis of primary adrenal insufficiency [17] and should be performed to confirm diagnosis in patients with clinical signs and symptoms of primary adrenal insufficiency, unless the results of basal cortisol levels are indicative of adrenal insufficiency [18]. Accordingly, it is recommended that therapy is started in patients with severe symptoms of adrenal insufficiency or adrenal crisis, without waiting for the result of a confirmatory test [2, 12, 16]. Presently, there is no laboratory marker available to confirm adrenal crisis, and diagnosis is based on the clinical presentation of the patient [9]. The diagnosis of Addison's disease in our case was based on a very low level of morning cortisol (0.84 *μ*g/dL) and the appearance of hyperpigmentation on the skin and mucosa, which is the classical clinical finding of primary adrenal insufficiency resulting from elevated levels of ACTH. Confirmatory examination with corticotropin stimulation test could not be performed because of limitations in laboratory facilities and this was not lead to any delay to start administering glucocorticoid treatment in our patient. The immediate administration of intravenous hydrocortisone was followed by significant clinical improvement, which also supports the diagnosis of primary adrenal insufficiency in our patient.

Other laboratory or radiological examinations have a limited role in confirming the diagnosis of adrenal insufficiency but have a greater role in evaluating its etiology. Determining the etiology or underlying conditions of adrenal insufficiency is the next step after confirming primary adrenal insufficiency based on hormonal levels [2]. The most common cause is autoimmune adrenalitis, either as isolated autoimmune adrenalitis or as a part of a group of autoimmune disorders known as autoimmune polyglandular syndrome, which is clinically classified into type 1 (APS-1) and type 2 (APS-2). Autoimmune adrenalitis in the case of APS-1 is associated with other immune disorders, such as hypoparathyroid, chronic mucocutaneous candidiasis, and other less common disorders (Hashimoto's thyroiditis, vitiligo, and premature ovarian failure), while in the case of APS-2, autoimmune adrenalitis is associated with hypothyroidism, type 1 diabetes mellitus, premature ovarian failure, and pernicious anemia. All of these disorders should be fully evaluated before confirming the diagnosis of isolated autoimmune adrenalitis. However, infection remains a common underlying cause in developing countries. Other possible etiologies that should be evaluated are medication use and malignancy [1, 5]. While ketoconazole, an antifungal agent, is known to possibly induce adrenal insufficiency, little is known about the effect of administering fluconazole [19]. Several case reports have demonstrated the evidence of a temporal reversible relationship between adrenal insufficiency (cosyntropin test) and the administration of high-dose intravenous fluconazole in critically ill patients [19]. Our patient received 150 mg of oral fluconazole every 24 h for the treatment of oral candidiasis, which was improved after 4 days of fluconazole doses. Administration of fluconazole in our case was unlikely an additional factor that exacerbated adrenal insufficiency, as significant clinical improvement was not observed after discontinuing fluconazole but by administering hydrocortisone.

Investigation of possible etiology in our case helped to exclude HIV infection, tuberculosis (based on chest X-ray examination), malignant tumors, or use of medication, although other autoimmune diseases associated with APS were not fully evaluated (hypoparathyroidism, autoimmune thyroid disease, and primary ovarian failure). Based on available practice guidelines, the evaluation of possible etiology or underlying conditions was still regarded as best practice (unrated evidence) [2]. Possible underlying conditions can be evaluated through serial examinations following the initiation of glucocorticoid therapy, based on symptoms that may appear later. Evaluation of other autoimmune diseases in our case were more focused on APS-2, since patients with APS-1 commonly have chronic mucocutaneous candidiasis since childhood, and APS-2 has a higher prevalence than APS-1 and more often progresses to adrenal crisis [5, 20]. Further evaluation of the possibility of primary ovarian failure in our case was planned by checking the level of follicle stimulating hormone, luteinizing hormone, and prolactin on the next outpatient visit; however, these hormone levels had not been evaluated before reporting this case.

Glucocorticoid therapy is recommended in all patients with primary adrenal insufficiency [2]. The suggested regimen is 15–25 mg of hydrocortisone or 20–35 mg of cortisone acetate in two or three divided oral doses per day. An alternative regimen is prednisolone (3–5 mg/day) administered orally in one or two doses. Dexamethasone should not be used in the case of primary adrenal insufficiency because of the risk of cushingoid effect, due to difficulties in titrating dexamethasone doses [2]. Adjusting the dose of hydrocortisone to body surface area (5.5 mg/m^2^) or body weight (0.12 mg/kg) may result in more physiological cortisol levels in primary adrenal insufficiency patients than fixed dose regimens [2]. Patients who present with critical illness or adrenal crisis should be administered parenteral hydrocortisone at an initial dose of 100 mg along with fluid resuscitation with isotonic saline. The dose of parenteral hydrocortisone is continued with 200 mg of hydrocortisone administered as continuous infusion over 24 h. On the next day, hydrocortisone is administered intravenously (100 mg every 24 h). Switching to oral preparation is based on the clinical improvement of the patient. Monitoring of therapy and adjustment of oral dose are also based on clinical presentations, not done by repeating hormonal evaluation [2]. The patient in our case had refractory shock that could not be stabilized by continuous infusion of norepinephrine, which led to the diagnosis of adrenal crisis. Therefore, we decided to administer intravenous hydrocortisone (100 mg every 24 h) and 30 min of continuous infusion of 100 ml normal saline for 7 days until hemodynamic and clinical symptoms were improved. Supportive therapy with fluid resuscitation was also administered to the patient. Following clinical symptom improvement, intravenous hydrocortisone was switched to an oral preparation, 20 mg in the morning and 10 mg in the afternoon, to resemble physiological cortisol secretion. Due to patient's good clinical response with oral hydrocortisone, the dose was reduced to single dose of 20 mg hydrocortisone in the morning. Cortisol in twice-daily dose gives satisfactory response in most patients; however, some patients may require only a single morning dose to maintain well-being and normal energy levels [1]. Outcome study of glucocorticoid therapy in the patient with adrenal insufficiency also found that less-frequent dosing may be associated with better compliance [21].

In conclusion, adrenal crisis should be considered in patients presenting with unexplainable vascular collapse. Presence of skin and/or mucosal hyperpigmentation would suggest primary adrenocortical insufficiency as underlying condition. Examination of basal cortisol level and ACTH test are recommended to diagnose Addison's disease. However, treatment with glucocorticoid should be started in patients with severe symptoms of adrenal insufficiency or adrenal crisis, without waiting for the result of a confirmatory test.

## Data Availability

The data used to support the findings of this study are available from the corresponding author upon request.
